# Identification of a pleiotropic QTL and candidate genes underlying ear traits of maize (*Zea mays* L.) through controlling the activity of inflorescence meristem

**DOI:** 10.1186/s12864-026-13186-9

**Published:** 2026-07-21

**Authors:** Xiaomeng Shen, Yifei Wang, Luyuan Wang, Yan Bai, Fenglin Jiang, Luling Jiang, Xiangyin Hou, Jinsheng Lai, Liru Cao, Weibin Song

**Affiliations:** 1 The Shennong Laboratory/Institute of Crop Molecular Breeding, Henan Academy of Agricultural Sciences, Zhengzhou, Henan 450002 China; 2 Department of Plant Genetics and Breeding, State Key Laboratory of Maize Bio-breeding, Key Laboratory of Genome Editing Research and Application, Ministry of Agriculture and Rural Affairs, National Maize Improvement Center, College of Agronomy and Biotechnology, China Agricultural University, Beijing, 100193 China; 3 National Agricultural Technology Extension & Service Center, Beijing, 100125 China; 4 Center for Crop Functional Genomics and Molecular Breeding, China Agricultural University, Beijing, 100193 P. R. China; 5 International Maize Research Center, Sanya Institute of China Agricultural University, Sanya, Hainan 572024 P. R. China

**Keywords:** Maize, Ear, Inflorescence meristem, Quantitative trait locus (QTL)

## Abstract

**Background:**

The ear of maize is a highly domesticated organ that has undergone a substantial increase in volume compared to its wild ancestor teosinte, resulting in a significant yield enhancement in modern cultivated maize. Application of quantitative trait loci (QTLs) mapping using the maize-teosinte segregation population facilitated the identification of more potential favorable variations for utilization in cultivation. However, the mature ear traits of the maize-teosinte population are influenced by genetic introgression from teosinte, which poses significant challenges to the investigation of yield-related traits due to abnormal sex differentiation of the inflorescence at the immature ear stage.

**Results:**

This study investigated mature ear traits along with the activity of the female inflorescence meristem (IM) using a Teosinte‑Maize introgression (TM) population via measuring the IM size. A total of 32 QTLs were detected across multiple environments over several years, with individual QTLs explaining 5.18% to 21.48% of the phenotypic variation. Notably, a major pleiotropic QTL regulating IM size and many ear traits was identified and narrowed to a 3.64 cM genetic interval on chromosome 3, namely qIM3. One candidate gene encoding *ZmEREB147* (*Zm00001d043205*) was identified with the help of transcriptome data, and Gene Ontology (GO) analysis demonstrated that qIM3 is involved in several hormone-mediated pathways and meristem development.

**Conclusions:**

The rapid implementation of these findings in maize molecular breeding holds important potential application value and provides broad prospects for improving maize yield.

**Supplementary Information:**

The online version contains supplementary material available at https://doi.org/10.1186/s12864-026-13186-9.

## Background

 Maize (*Zea mays* L.), a globally significant cereal crop, was domesticated approximately 9,000 years ago from its wild progenitor, teosinte (*Zea mays ssp. parviglumis)* [1, 2]. Domestication and subsequent selective breeding have driven profound divergence in vegetative and inflorescence architecture between maize and teosinte, with particularly striking differences observed in the female inflorescence, also called ear. Teosinte exhibits a highly branched architecture, characterized by elongated lateral branches that produce multiple small ears along the internodes and a terminal tassel at the branch apex. In contrast, modern cultivated maize has an erect, unbranched main stem bearing a single fully developed ear and a terminal tassel, with axillary branching and tillering largely suppressed. These differences in plant and ear architecture reflect fundamental changes in meristem fate, axillary meristem activity, and ear developmental programs during maize domestication [3–5]. Domestication altered morphological traits and reshaped genome architecture, including the loss or fixation of beneficial alleles in wild progenitors, which now serve as valuable genetic resources for crop improvement [6–8]. Accumulating evidence suggests that teosinte harbors numerous favorable alleles absent in maize at several quantitative trait locus (QTLs). By using Teosinte-NAM population (TeoNAM) or the BC_2_S_3_ recombinant inbred line populations (RILs) for QTL mapping, several key loci and genes controlling domestication-related traits have been identified. Notably, *ZmCCT10* [9, 10] and *ZmCCT9* [11] regulate flowering time; The mutation of the *FEA4* gene underlying QTL *TBN6.1* causes fasciated ears and tassels [12], whereas *TSH4*, corresponding to QTL *TBN7.2*, controls lateral organ growth and affects inflorescence development [13]. Additionally, introgression of wild alleles such as *UPA2*, which were lost during maize domestication, into modern hybrids together with genome editing of *ZmRAVL1* to alter leaf angle, has been demonstrated to optimize plant architecture and enhance yield under high-density planting conditions [14–16]. Therefore, systematically utilizing favorable alleles from teosinte-derived QTL provides a promising strategy for improving yield and breeding efficiency in modern maize.

Maize ear traits, including ear length (EL), kernel number per row (KNPR), cob diameter (CD), kernel row number (KRN), and kernel weight (KW), are typical quantitatively inherited traits controlled by multiple genes and serve as key determinants of yield [17–21]. The quantity of these yield-related traits is regulated by the initiation and development of axillary meristems (AMs), which originate from the shoot apical meristem (SAM), and is strongly influenced by the ear morphogenesis [22–24]. During the transition from vegetative to reproductive growth, IM activity – which derives from AM differentiation – becomes critical, as it significantly influences ear morphogenesis and development [25–27]. Within the IM, the dynamic balance between stem cell renewal and organ initiation governs branch formation, spikelet differentiation and floret number [28–30]. Consequently, IM size and activity directly influence kernel differentiation, thereby affecting key yield-related traits [31–34]. Precise regulation of IM activity and size is therefore fundamental to determining ear architecture and overall yield potential in maize.

Extensive studies have identified numerous genes regulating maize ear development, revealing a complex molecular network encompassing the CLV-WUS feedback pathway, RAMOSA pathway, miRNA signaling, and regulation by multiple plant hormones, including auxin, cytokinin, gibberellin, and ethylene [35, 36]. In maize, the CLV-WUS pathway plays a decisive role in ear morphology by controlling SAM and IM activity and size [23–39]. *ZmWUS1* and *ZmWUS2* maintain stem cell fate in the IM [40, 41], whereas *TD1* [42], *FEA2* [43, 44], *FEA3* [45], the bZIP transcription factor gene *FEA4* [12] and *GRXs* [46] fine-tune meristem activity by perceiving CLE peptide signals and regulating meristem zonation and fasciation. Mutants with enhanced IM activity can alter kernel row number and lead to flattened ears, including *fea2*, *fea3*, *fea4*, *grxs*, and *zmcrn*. Moreover, in weak alleles of *FEA2* and *FEA3*, a moderate increase in IM activity results in increased EL, KRN, KNPR, and EW, thereby enhancing yield per ear [31, 45]. Studies have demonstrated that plant hormones are important regulators of meristem activity: auxin regulates axillary meristem formation, with *spi1* mutants showing reduced spikelet primordia and lower kernel number [47]; gibberellin mutants *vt2* exhibit similar developmental defects [48]; ethylene regulates ear length, spikelet number, and fertility, with *qEL7*/*ZmACO2* affecting ethylene biosynthesis in developing ears [49]. Other essential genes, including *BA1*, *BA2*, *BAF1*, and *BIF2*, are required for ear initiation and branching, with *BIF2* directly controlling auxin transport via *ZmPIN1a* [50–53].

Although numerous QTL associated with maize ear traits have been identified across diverse populations, only a few have been successfully cloned, and even fewer function stably in hybrid backgrounds. Therefore, using segregating populations derived from crosses between maize and its wild ancestor teosinte provides an effective approach to dissect IM morphology and ear-related traits when combined with genetic mapping. This approach elucidates the genetic regulation of IM activity and uncover key yield-related genes lost during domestication and improvement. This study focuses on the female inflorescence meristem, examining how IM activity and size influence spikelet primordia differentiation and, consequently, ear architecture and other agronomic traits. Identifying and characterizing genes and loci that regulate IM activity will deepen our understanding of maize ear development and yield formation, while providing valuable targets for molecular breeding to enhance maize productivity.

## Materials and methods

### Plant materials and field trials

This study developed a maize–teosinte population by backcrossing the modern elite maize inbred line Zheng58 (recurrent parent) with teosinte (*subspecies parviglumis*), followed by selfing to the BC₂F₂ generation. The population comprised 590 lines and was defined as the Teosinte-Maize introgression (TM) population. TM was cultivated in 2022 in Tieling, Liaoning Province (42.30°N, 123.86°E), and in 2024 in Xinxiang, Henan Province (35.30°N, 113.93°E), and in 2024 in Sanya, Hainan Province (18.28°N, 109.51°E), with two replicates per environment. Each family was planted in a single-row plot (13 plants per row, 60 cm row spacing, 25 cm plant spacing). Standard water and fertilizer management practices were applied throughout the maize growth period to meet developmental stage-specific requirements.

During the 5 mm stage of ear development (defined as the stage when the entire ear length from its base to tip reaches 5 mm), samples were collected for inflorescence meristem (IM) analysis. The morphology and size of the IM were observed using a stereomicroscope, with dimensions recorded as height (IMH) and width (IMW). IMH and IMW were defined as the maximum height and width of the IM tissue, measured from ear tip to the apex of the suppressed bracts (SB). At maturity, ear-related traits in the population were evaluated, including ear row number (KRN), ear length (EL), ear diameter (ED), ear weight (EW), and kernel weight (KW). EL was measured from the tip to the base of the ear, and ED was assessed at approximately two-thirds of the ear length from the base. For each line, 6–8 juvenile and mature ears were evaluated, measured, and recorded for statistical analysis. We performed an additional experiment with a randomly diverse panel of 117 inbred lines. We measured the ear length of mature ears, and three biological replicates were measured per line, with each replicate consisting of 6–8 ears.

### Statistical methods of phenotypes

Descriptive statistics were analyzed using IBM SPSS Statistics (v26.0). Raw phenotypic data were initially organized in Microsoft Excel 2024 (Microsoft Corporation, Redmond, WA, USA) before being imported into R (version 4.4.0; https://www.r-project.org) for subsequent statistical analyses. The trait means for each line are summarized in Table S1 and Table S2 (Supplemental Material). Broad-sense heritability (H²) for KRN, ED, EL, IMH and IMW was calculated according to the method described in reference [54] using the formula: H^2^ = δ^2^ g / (δ^2^ g + δ^2^ e / n), where δ^2^ g represents genetic variance, δ^2^ e denotes residual variance, and n indicates the number of environments. Variance components δ^2^ g and δ^2^ e were estimated via a mixed linear model implemented in the R package lme4, with genotype, environment, and replication treated as random effects. The statistical significance of variance components was evaluated using the lmerTest package. To account for environmental variation in QTL detection, best linear unbiased predictors (BLUPs) for each line across the two environments were computed using the same mixed linear model implemented in lme4, fitting both genotype and environment as random effects [54]. BLUP values of the TM population were combined for subsequent QTL mapping and downstream analyses. Phenotypic data were imported using the readxl and readr packages and cleaned, filtered, and reshaped using dplyr and tidyr. Pearson correlation coefficients among ear-related traits were computed using the Hmisc package, and pairwise correlation matrices and scatterplot matrices were generated using GGally. Data visualization was performed using ggplot2, with numeric scaling and axis formatting implemented using the scales package. Multi-panel figures were assembled using cowplot, gridExtra, and grid. Both raw phenotypic values and BLUP-adjusted values were used for all subsequent genetic analyses, including QTL mapping.

### Linkage map construction and QTL identification

Genotypic data for the TM population at the BC₂F₂ generation were obtained through a single round of whole-genome resequencing. Clean reads were aligned to the B73_V4 reference genome using BWA. Variant calling was performed for each sample using the GATK v4.2.6.1 HaplotypeCaller to generate individual GVCF files, which were subsequently jointly genotyped across all samples to produce a multi-sample VCF file [55]. Quality control was performed on the VCF variant dataset by applying the following criteria: SNPs with a minor allele frequency (MAF) < 0.05 were removed; individuals with a SNP missing rate greater than 50% were excluded; and SNPs with a missing rate exceeding 40% were filtered out [56]. After these steps, a high-quality population-level SNP variant dataset was obtained. Genetic linkage maps were constructed using the R package “R/qtl” [57], followed by identification of QTLs for ear-related traits in the population. Genotype data were imputed using the “fill.geno” function, and linkage maps were generated with the “est.map” function, employing the “Kosambi” recombination model. QTL mapping was performed using the Composite Interval Mapping (CIM) method, with significance thresholds determined by 1,000 permutations (LOD = 3, *P* < 0.05). QTL confidence intervals were defined as regions spanning 1.5 LOD units below the peak LOD score.

### Transcriptome sequencing and differential gene expression analysis

After QTL mapping, two near-isogenic lines (NILs) of *qIM3* (qIM3^Z58^ and qIM3^Teo^) were identified with difference in IM phenotype and harboring segments of Zheng58 and teosinte in *qIM3* region, respectively. Seeds were sown and IM samples were collected at the 5 mm stage of ear development. For each line, 3–4 ear samples were harvested and immediately frozen in liquid nitrogen. Additionally, we randomly collected IM samples from 117 inbred lines using the same sampling method (two biological replicates per line). Samples were sent to BGI Life Sciences (Sanya, China) for RNA extraction and transcriptome sequencing. RNA quality was assessed using Qsep100 and Qubit 4.0, and mRNA sequencing libraries were constructed using the strand-specific RNA-Seq protocol. Sequencing was performed on the DNBSEQ™ platform (BGI). Data analysis included alignment of reads to the B73_v4 reference genome (https://download.maizegdb.org/Zm-B73-REFERENCE-GRAMENE-4.0) [58] using Hisat2 (v2.2.1) [59], followed by gene expression quantification with StringTie (v2.1.4) [60] based on uniquely mapped reads. Gene expression levels were normalized using “DESeq2” package in R (v1.26.0) [61]. Gene Ontology (GO) enrichment analysis was conducted using AGRIGO v.2.0 (http://systemsbiology.cau.edu.cn/agriGOv2) [62], and GO terms with adjusted *P* < 0.01 were considered significantly enriched. The RNA-seq dataset for gene expression variation study of *ZmACO2* was downloaded from the NCBI SRA database (https://www.ncbi.nlm.nih.gov/bioproject/PRJNA705033) by SRR13787130 - SRR13787135 [49].

## Results

### Phenotype evaluation of the mapping population

This study utilized the TM population comprising 590 inbred lines derived from Zheng58 introgressed with teosinte as experimental materials. Phenotypic evaluations of ear-related traits, including ear length (EL), ear diameter (ED), kernel row number (KRN), kernel weight (KW), and ear weight (EW), were conducted across multiple environments(in Tieling, Sanya and Xinxiang) in 2022 and 2024. In 2022TL and 2024SY, the mean EL values were 13.9 ± 2.4 cm and 10.7 ± 1.7 cm, with corresponding ranges of 7.4 to 18.3 cm and 6.4 to 14.3 cm, respectively. ED measurements were 33.9 ± 4.2 mm in 2022TL and 33.7 ± 4.3 mm in 2024SY, ranging from 24.2 to 42.5 mm and from 19.6 to 42.3 mm, respectively. KRN exhibited mean values of 11.0 ± 1.3 and 10.9 ± 1.3 across the two environments, with variation ranges of 8.0 to 13.8 and 7.7 to 13.6, respectively (Table S1). In 2024SY, the mean KW was 33.4 ± 12.7 g, with values ranging from 7.5 to 103.7 g. The EW showed mean values of 49.0 ± 18.9 g, with variation ranges of 11.9 to 137.8 g (Table S2). These results demonstrate substantial phenotypic variation for ear-related traits within the population.

**Fig. 1:**
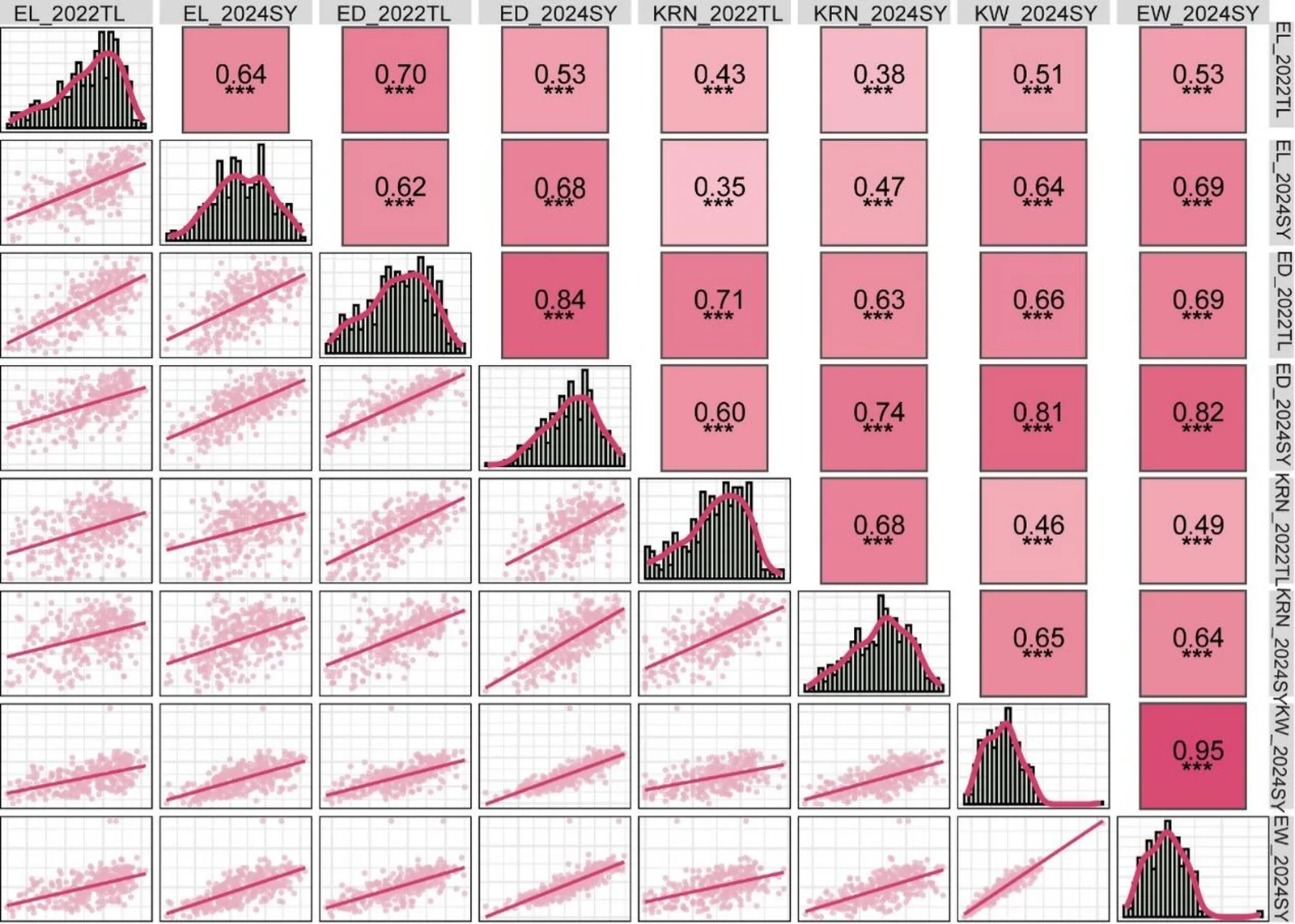
The frequency distributions and Pearson correlation coefficients of Ear Length (EL), Ear Diameter (ED) and Kernel Row Number (KRN) of TM population in different environments in 2022 Tieling, 2024 Sanya, Ear Weight (EW) and Kernel Weight (KW) in 2024 Sanya were plotted. ***, *P* < 0.001

To assess the genetic basis of these traits, broad-sense heritability (H²) was estimated using a mixed linear model. Among all traits, ED exhibited the highest heritability (H² = 0.85), followed by KRN (H² = 0.69) and EL (H² = 0.59) (Table S1), suggesting that these traits are predominantly governed by genetic factors. The phenotypic distributions of all ear-related traits were continuous and approximately normal across environments (Fig. 1).

Correlation analyses were subsequently conducted to examine relationships among ear-related traits across environments (Fig. 1). High correlations were observed for the same trait between environments, with the correlation coefficient between ED_2022TL and ED_2024SY reaching 0.84. In addition, all ear-related traits exhibited significant positive correlations with each other, with generally high correlation coefficients. Strong correlations were detected between ED_2022TL and EL_2022TL (*r* = 0.70), as well as between EW_2024SY and ED_2024SY (*r* = 0.82). The correlation between ED and EL, as well as between ED and EW, is influenced by the interactions of spatial and nutritional factors during development, demonstrating a certain level of mutual constraint. In contrast, the correlations between ED, KRN, and EL imply that these traits may be governed by shared genetic regulatory mechanisms. Together, these findings suggest that maize ear yield-related traits could be co-regulated during ear development, offering a strong foundation for future joint QTL analyses and integrated breeding strategies.

### Construction of high-density linkage map and physical map

All ten maize chromosomes were covered by high-density molecular markers, which exhibited a relatively uniform distribution (10,330 markers spanning 1,160 cM of genetic distance, with an average interval of 0.11 cM between markers) (Table 1). No significant marker gaps or extreme segregation distortion were detected. Overall, Zheng58 and *Zea mays ssp. parviglumis* alleles exhibited an alternating mosaic pattern along the chromosomes, indicating abundant recombination events following two rounds of backcrossing. Genotype distribution analysis of the BC₂F₂ population revealed a lower proportion (10.9%) of parviglumis alleles, with Zheng58 alleles predominating at 89.1%. The population therefore shows a stable genetic structure and balanced recombination, providing sufficient genetic variation for subsequent QTL analyses.

**Table 1:** The introgression proportion of teosinte fragments in TM population

Chr^a^	Markers^b^	Length (cM)^c^	Maize (%)^d^	Heterozygous (%)	Teosinte (%)^e^
1	1917	157	78.32	21.20	0.48
2	1134	147	83.81	15.33	0.86
3	1324	103	75.83	22.59	1.58
4	632	142	88.27	11.24	0.49
5	1164	132	80.56	17.51	1.93
6	639	104	84.47	13.20	2.33
7	929	102	76.27	17.38	6.35
8	1098	94	80.25	17.07	2.68
9	849	91	79.07	13.70	7.23
10	644	88	84.23	10.92	4.85

^a^Chr: Chromosome

^b^Markers: Total number of markers

^c^Length: Total genetic distance of this chromosome

^d^Maize (%): The genomic proportion of Zheng58 in the TM population across the current chromosome

^e^Teosinte (%): The genomic proportion of *Zea mays ssp. Parviglumis* in the TM population across the current chromosome

### QTL analysis using linkage mapping in TM population

**Fig. 2:**
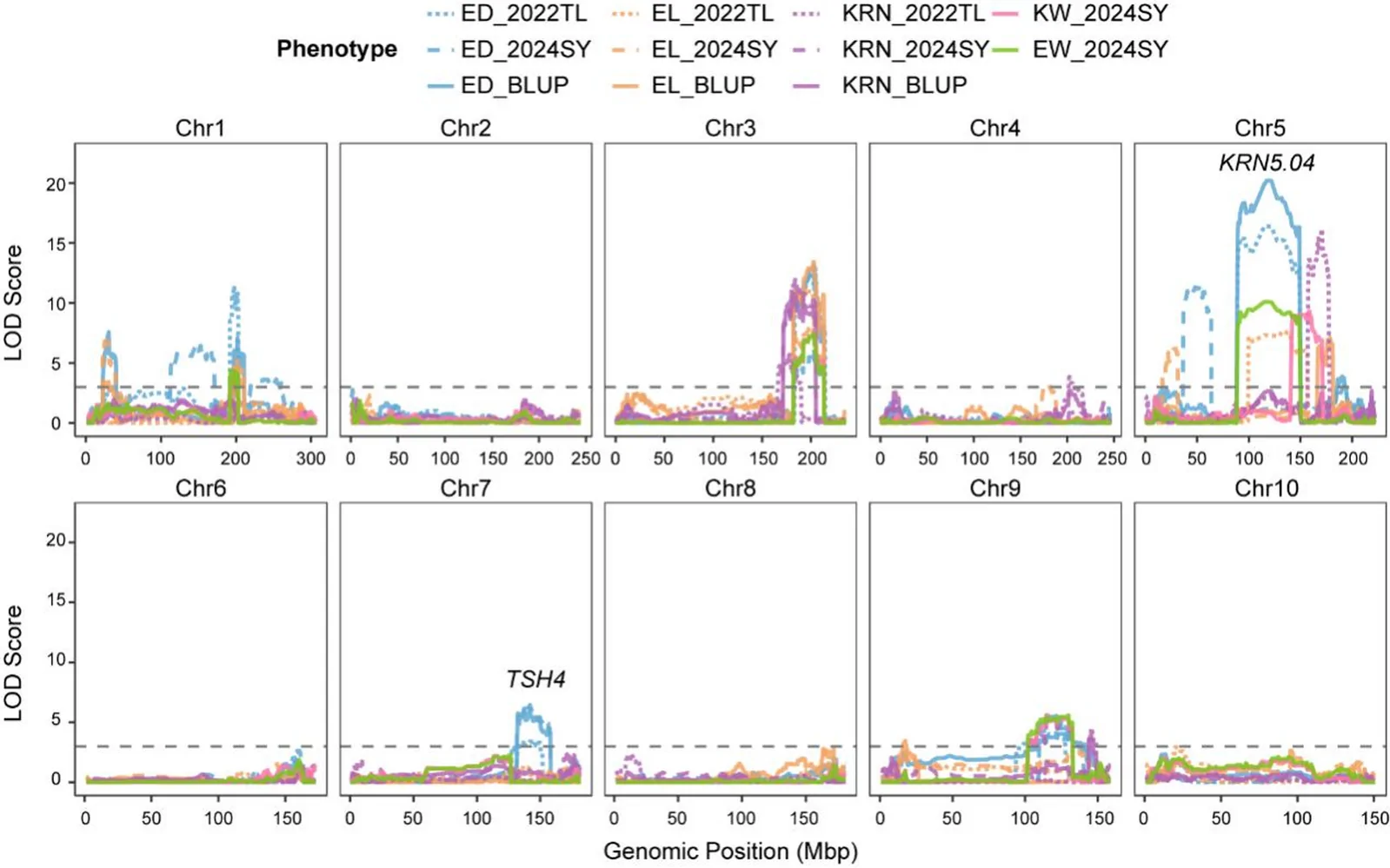
QTL mapping results of ear length (EL), ear diameter (ED), kernel row number (KRN), kernel weight (KW) and ear weight (EW) in 2022TL, 2024SY and BLUP, as well as known cloned genes located within the QTL intervals identified on different chromosomes, including *Kernel Row Number 5.04 (KRN5.04)*, and *TASSELSHEATH4 (TSH4)*

To further elucidate the genetic basis of yield traits in the TM population, we performed a genome-wide QTL scan for ear-related traits. A total of 21 QTL detection events associated with five yield-related traits, including EL, ED, KRN, EW, and KW, were identified across different environments (2022TL and 2024SY trial sites, as well as BLUP analysis). Because several QTLs were co-localized across multiple traits, these detection events were ultimately integrated into 13 unique QTL loci. These QTLs were distributed across all ten maize chromosomes, with three QTLs for KRN located on chromosomes 3, 5, and 9; six EL-related QTLs on chromosomes 1, 3, and 5; eight ED-related QTLs on chromosomes 1, 3, 5, 7, and 9; and two QTLs for KW on chromosomes 3 and 5; and two QTLs for EW on chromosomes 3 and 5 (Fig. 2; Table 2). Among these, individual QTLs for EL showed phenotypic contribution rates ranging from 6.52% to 15.34%, with additive effects ranging from 0.61 to 2.12 cm. For ED, the contribution rate ranged from 5.42% to 19.69%, with additive effects ranging from 2.32 to 4.84 mm. For KRN, the phenotypic contribution ranged from 5.18% to 21.48%, with additive effects ranging from 0.73 to 1.52 row.

**Table 2:** Consensus QTLs for ear length (EL), ear diameter (ED), kernel row number (KRN) in 2022TL, 2024SY and BLUP, kernel weight (KW) and ear weight (EW) in 2024SY

Trait name	QTL	Environment	Chr^a^	LOD^b^	Var(%)^c^	Add^d^	Peak (Mb)^e^	Support interval (Mb)^e^	Peak_Pos (cM)^f^
KRN	*qKRN3*	BLUP	3	10.76	11.35	0.73	183.51	173.68-188.28	63.53
	*qKRN3*	2022TL	3	5.69	6.78	0.75	177.90	169.13-180.31	60.46
	*qKRN3*	2024SY	3	12.28	13.62	1.12	184.42	179.53-188.28	64.16
	*qKRN5*	2022TL	5	16.17	21.48	1.52	170.22	165.52-171.77	76.56
	*qKRN9*	2024SY	9	5.25	5.18	0.79	115.83	113.79-133.16	43.43
EL	*qEL1.1*	BLUP	1	3.43	8.07	0.61	30.03	21.69–39.46	31.99
	*qEL1.1*	2024SY	1	6.82	6.52	0.94	24.95	22.00-30.97	29.74
	*qEL1.2*	2022TL	1	5.19	7.67	1.79	201.80	196.23-211.23	93.25
	*qEL3*	BLUP	3	13.43	15.34	1.20	202.33	192.43-205.14	68.09
	*qEL3*	2022TL	3	11.18	14.73	2.12	197.66	191.44-205.14	67.64
	*qEL3*	2024SY	3	8.82	11.06	1.17	202.40	192.43-205.14	68.09
	*qEL5.1*	BLUP	5	7.10	7.41	0.95	178.38	174.86-181.64	82.44
	*qEL5.2*	2022TL	5	7.65	9.11	2.07	131.71	99.14-146.36	64.89
	*qEL5.3*	2024SY	5	6.20	6.60	1.11	29.35	19.11–31.57	38.73
ED	*qED1.1*	BLUP	1	7.58	12.57	2.32	30.22	26.52–30.97	32.08
	*qED1.2*	2022TL	1	11.43	10.20	3.60	197.70	194.61–200.20	89.24
	*qED1.3*	2024SY	1	6.63	9.48	2.41	146.60	116.91-172.61	67.8
	*qED3*	BLUP	3	13.15	12.95	2.83	202.33	194.24-203.52	68.09
	*qED3*	2022TL	3	12.86	13.47	3.61	204.48	194.24-205.43	68.44
	*qED3*	2024SY	3	6.36	8.69	2.88	202.40	197.01-205.43	68.09
	*qED5.1*	BLUP	5	20.22	15.16	2.62	117.52	103.97-132.64	63.44
	*qED5.1*	2022TL	5	16.42	19.69	4.84	115.21	103.97-137.92	63.24
	*qED5.2*	2024SY	5	11.45	12.04	2.81	53.68	37.83–59.82	47.05
	*qED7*	BLUP	7	6.15	5.88	2.45	142.17	131.86-150.65	60.54
	*qED7*	2024SY	7	6.43	5.90	3.12	139.71	131.86-151.97	59.77
	*qED9*	2024SY	9	5.91	5.42	3.11	124.8	109.47-124.86	46.38
KW	*qKW3*	2024SY	3	7.74	10.34	8.24	202.33	191.81-205.14	68.09
	*qKW5*	2024SY	5	9.28	11.48	10.23	158.89	139.74–161.60	72.28
EW	*qEW3*	2024SY	3	7.48	8.58	11.05	202.33	191.81-205.14	68.09
	*qEW5*	2024SY	5	10.10	12.15	15.27	115.21	91.11-146.36	63.24

^a^Chr: Chromosome

^b^LOD: LOD means logarithm of odds

^c^Var (%): Var indicates the percentage of phenotypic variation explained by QTL

^d^Add: Positive or negative addictive effect values indicates that the alleles from Zheng58 or Teosinte increased the phenotypic value, respectively. The units for EL, ED, KW, and EW are centimeters (cm), millimeters (mm), and grams (g), respectively

^e^Mb: Physical positions are given in megabases (Mb) based on the B73 RefGen_v4 genome assembly

^f^Peak_Pos (cM): The position of the peak for each quantitative trait locus (QTL) on the genetic map, measured in centimorgans (cM)

Based on the results, QTLs for KW, EW, and EL were identified on chromosome 5, within which the reported gene *KRN5.04* plays a role in negatively regulating KRN and has a moderate effect on 10-kernel width, 100-kernel weight, kernels per ear, and grain yield per ear [63]. Additionally, a QTL for ED located on chromosome 7 was found within the region containing *TSH4* [13], which encodes an SBP-box transcription factor involved in inflorescence boundary regulation. Notably, a region on chromosome 3 was identified as a pleiotropic locus for EL, ED, KRN, EW, and KW. Individual QTLs showing logarithm of odds (LOD) scores ranging from 5.69 to 13.43 and phenotypic contribution rates from 6.78% to 15.34%, all demonstrating positive additive effects conferred by the Zheng58 allele. Within this novel QTL interval, no major QTL controlling ear development has been reported in previous studies, suggesting it may represent a new genetic locus controlling multiple ear traits. In summary, the identified QTLs in TM population provide valuable insights into the genetic regulation of ear development, offering a theoretical basis for the genetic improvement of yield traits such as EL, ED, and KRN in maize.

### *qIM3* regulates yield-related trait through its pleiotropic effect on meristem activity

In numerous individual plants of the TM population, the apical region of the female inflorescence becomes thinner at the approximately 5 mm stage and incorrectly differentiates into male floral structures. This leads to breakage and shedding without kernel formation at the ear maturity stage. This masculinization of maize ear in the TM population leads to inaccurate measurements of traits such as EL and KNPR, thereby amplifying the observed variation and reducing reproducibility in QTL mapping (Fig. S1). To address this issue, we adopted an alternative approach based on the understanding that early inflorescence development is controlled by the activity of the inflorescence meristem (IM), which is largely reflected in the meristem region volume and determines ear-related traits [22]. Given that IM activity reflects the potential for agronomic traits, we conducted observations and statistical analysis of IM size and performed QTL calculations. To address this, we measured the dimensions of the IM at the early developmental stage (~ 5 mm) of ear, including IM height (IMH) and IM width (IMW).

**Fig. 3:**
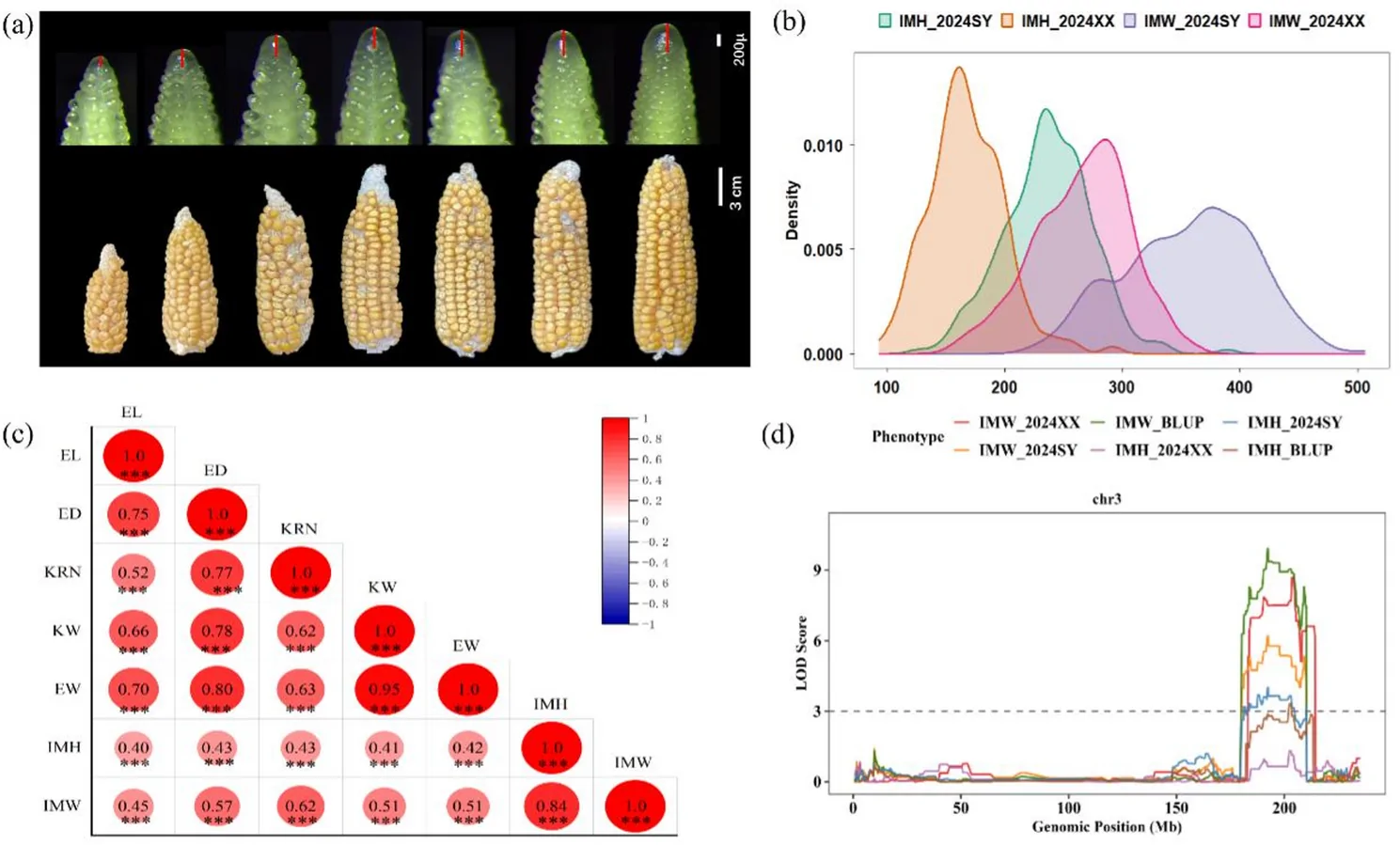
Phenotypic variation, correlation, and QTL mapping of IM- and ear-related traits in the TM population. **a** Variability in ear and inflorescence meristem (IM) morphology among individuals of different lines of the TM population. IM and ear samples were collected from different plants at vegetative and mature stages, respectively. Bars in (**a**) are 200 μm and 3 cm separately. **b** Phenotypic data distribution of IMH and IMW across different environments; IMH and IMW are measured in millimeters (mm); IM-W, inflorescence meristem width; IM-H, inflorescence meristem height. **c** Pearson’s correlation coefficients between seven ear traits (correlation coefficient > 0.3 and *P* < 0.001). **d** QTL mapping results for IMH and IMW on chromosome 3

In the 2024XX and 2024SY environments, the mean IMH values were 167.6 ± 32.0 μm and 236.9 ± 37.6 μm, respectively, with variation ranges of 93.0 to 291.6 μm and 126.4 to 389.2 μm; IMW values were 266.0 ± 40.0 μm and 356.9 ± 54.6 μm, with ranges of 158.0 to 358.0 μm and 221.9 to 506.3 μm (Fig. 3a, Fig. S2, Table S1). The broad-sense heritability (H²) of IMW was 0.55, and that of IMH was 0.28 (Table S1). The IMW and IMH values meets the threshold of above 0.2 required for QTL mapping, confirming that these two traits are under mainly genetic control for subsequent genetic analysis. Both IMH and IMW in different environments followed a continuous near-normal distribution (Fig. 3b), indicating that IM dimensions are quantitatively inherited. Correlation analysis between the measured IM dimensions and mature ear traits, including EL, ED, KRN, EW, and KW, revealed significant correlations (Fig. 3c). IM activity reflects the proliferative capacity of meristem cells, which drives increases in both vertical and horizontal tissue architecture. Meristem volume is usually used to describe its activity. The correlation between IMH and IMW is 0.84, which were treated as two size indicators used for volume statistics, suggesting that IM size is tightly coupled during inflorescence meristem development. Both dimensions correlated significantly with ear traits, with IMW showing stronger associations than IMH, particularly with KRN. The size of the inflorescence meristem (IMH and IMW) correlated significantly with ear length (EL), highlighting its strong association with ear length formation. We observed that IM size variation is closely associated with the meristem’s ability to progress through normal development. These findings confirm that IM activity is crucial for determining maize ear morphology and yield during ear development.

To further investigate the genetic basis of IMW and IMH, and their association with ear traits, we performed QTL identification across two environments and BLUP analysis. A total of 11 QTLs were identified: five for IMW (on chromosomes 1, 3 and 5) and six for IMH (on chromosomes 1, 2, 3 and 5). For IMH, individual QTLs explained phenotypic variation ranging from 5.28% to 13.32%, with additive effects ranging from 6.20 to 33.72 μm. For IMW, individual QTLs explained phenotypic variation from 5.40% to 15.22%, with additive effects ranging from 15.55 to 40.10 μm (Table S6). Notably, overlapping QTLs for both IMW and IMH were identified on chromosomes 1, 3 and 5, with particularly high overlap on chromosome 3 (Fig. 3d). *qEL* overlapped with *qIMH3*, *qIMW3*, *qIMH1*, *qIMH5*.2, *qIMH5*.3 and *qIMW1.1*, *qKRN* shared common loci with *qIMH3*, *qIMW3* and *qIMW5.1*. *qED* co-localized with *qIMH3*, *qIMW3*, *qIMW1.1* and *qIMH5.3*, whereas both *qEW* and *qKW* overlapped with *qIMH3*, *qIMW3* and *qIMH5.3*. In addition, the QTLs *qIMW3*, *qIMH3*, *qEL3*, *qED3*, *qKRN3*, *qEW3*, and *qKW3* on chromosome 3 exhibited high co-localization and were combined and renamed as *qIM3* (located within a 3.64 cM genetic interval and a physical location spanning 189.71 to 205.14 Mb on chromosome 3) (Tables 2 and 3). These overlapping QTLs are consistent with the hypothesis that variation in ear-related traits is controlled by IM activity. Compared to the results obtained from mature trait, the QTLs identified for IM dimensions were more concentrated and precise, with narrower confidence intervals. These findings suggest that this pleiotropic locus may regulate meristem activity and thereby control the yield-related traits of the ear.

**Table 3:** Consensus QTLs for IMH and IMW in 2024XX, 2024SY and BLUP

Trait name	QTL	Environment	Chr^a^	LOD^b^	Var (%)^c^	Add^d^	Peak (Mb)^e^	Support interval (Mb)^e^	Peak Pos (cM)^f^
*IMH*	*qIMH3*	2024SY	3	4.01	5.68	20.02	192.36	179.53-207.13	66.68
*IMW*	*qIMW3*	2024XX	3	8.68	15.22	40.10	203.55	189.71-205.43	68.36
		2024SY	3	6.20	9.80	35.57	192.24	182.90-205.43	66.68
		BLUP	3	9.92	11.41	21.43	192.24	189.71-205.14	66.68

^a^Chr: Chromosome

^b^LOD: LOD means logarithm of odds

^c^Var (%): Var indicates the percentage of phenotypic variation explained by QTL

^d^Add: Positive or negative addictive effect values indicates that the alleles from Zheng58 or Teosinte increased the phenotypic value, respectively; IMH and IMW are measured in micrometers (µm)

^e^(Mb): Physical positions are given in megabases (Mb) based on the B73 RefGen_v4 genome assembly

^f^(cM): The position of the peak for each quantitative trait locus (QTL) on the genetic map, measured in centimorgans (cM)

### Candidate genes for *qIM3* and genetic regulatory network of inflorescence meristem activity

**Fig. 4:**
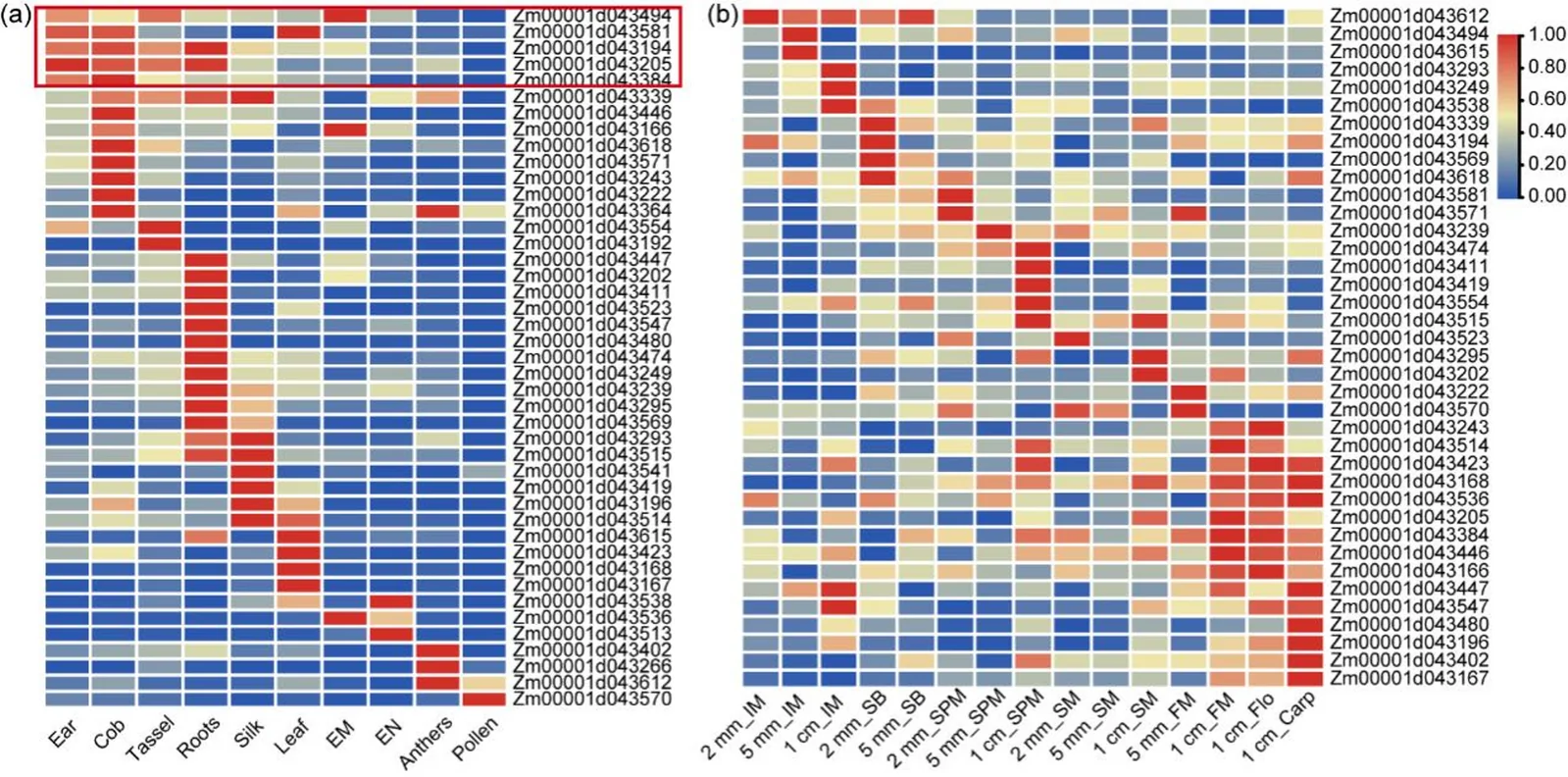
Relative expression of genes at qIM3 across ten tissues (Ear, Cob, Tassel, Roots, Silk, Leaf, Embryo, Endosperm, Anthers and Pollen) and in inflorescence tissues at different developmental stages (Shen et al., 2023). Red boxes indicate genes with relatively higher expression in the ear compared to other tissues

Since *qIM3* is a novel QTL with pleiotropic effects on ear traits, we targeted the selected region for further investigation. Two near-isogenic lines (NIL-qIM3^Z58^ and NIL-qIM3^Teo^) were selected for transcriptome sequencing. Genotypic differences were observed at the *qIM3* locus, while the genetic backgrounds at other identified QTL loci remained consistent. The two NILs showed 71.1% genetic background similarity genome-wide, with the same genotype at other QTLs identified in this study. Thus, these two materials can be considered near-isogenic lines, each containing distinct segments from maize Zheng58 and teosinte, respectively, at the *qIM3* region. A total of 163 ear-expressed genes (FPKM > 1) were identified in the *qIM3* region, among which 43 genes exhibited significant differential expression (|log2FC| > 0.585, padj < 0.05). Based on expression pattern analysis [64], 18 out of the 43 genes were found to be predominantly expressed in vegetative organs such as leaves, roots, and stems, or floral organs like silk, anthers, and pollen. These genes were subsequently filtered out as they were deemed unlikely to be major candidates for regulating ear development (Fig. 4). Consequently, 25 ear-highly-expressed differentially expressed genes (DEGs) were regarded as candidate genes in *qIM3* contributing to ear development.

Among the candidate genes, *ZmEREB147* (*Zm00001d043205*) exhibited high expression in ear, cob, tassel (log2FC = -0.647, padj < 0.05) and was also expressed in several inflorescence meristems. *ZmEREB147* was expressed higher in NIL-qIM3^Z58^ than NIL-qIM3^Teo^, with expression levels of 54.3 and 33.8 FPKM, respectively. NIL-qIM3^Z58^ exhibited a modestly larger IM size compared to NIL-qIM3^Teo^ (Fig. S2), indicating that *ZmEREB147* may positively regulate the inflorescence meristem. *ZmEREB147* encodes a nuclear-localized AP2/ERF transcription factor. Members of this family response to the ethylene signaling and are implicated in meristem fate determination [65]. Another DEG, *SBT22* (*Zm00001d043581*), which showed elevated expression in ear tissue, encodes a subtilisin-like protease (log2FC = -0.721, padj < 0.05). The subtilisin-like protease is potentially involved in peptide signal maturation and intercellular communication, suggests a correlation with the CLV-WUS signaling pathway [66]. Additionally, *Zm00001d043494* is expressed at higher levels in IM region, implying a potential role in meristem activity or early developmental processes (log2FC = -0.586, padj < 0.05).

To further elucidate the regulatory pathways of *qIM3* in controlling IM activity, Gene Ontology (GO) enrichment analysis was performed using DEGs from the entire genome between the NILs with different alleles of *qIM3* (NIL-qIM3^Z58^ and NIL-qIM3^Teo^). The *ZmEREB147* up-regulated genes were mainly enriched in regulation of meristem growth (GO:0010075; FDR = 0.038). In contrast, the *ZmEREB147* down-regulated genes were significantly enriched in response to chemical (GO:0042221; FDR = 0.0084). Among the identified GO terms, the ethylene-related ones fall under the category of the GO term response to chemical. Notably, *ZmEREB147* is an ethylene-responsive transcription factor. This implies that the expression variation of *ZmEREB147* within the *qIM3* locus may contribute to phenotype by regulating differentially expressed genes involved in meristem development. Thus, *ZmEREB147* may act as a key node, translating chemical and metabolic cues into the transcriptional signals that direct inflorescence meristem development.

## Discussion

Since its domestication from the wild ancestor teosinte (*Zea mays ssp. parviglumis*), maize has undergone extensive artificial selection, resulting in profound changes in plant architecture, inflorescence structure, kernel traits, and flowering behavior. Among these traits, ear architecture has been a primary target of selection, as it directly determines grain yield and harvest efficiency. It has played a pivotal role during the transition from hunting-gathering societies to agriculture [67]. However, despite decades of research, only a limited number of ear-related domestication genes have been cloned and functionally characterized, including *TGA1*, which controls glume hardness, and *KRN2* and *KRN4*, which regulate kernel row number [34, 68–70]. This limited repertoire suggests that the genetic architecture underlying maize ear development remains incompletely understood. This also suggests that numerous critical regulators underlying ear morphogenesis and yield formation have yet to be uncovered.

In this study, we exploited a Teosinte-Maize introgression (TM) population to dissect the genetic basis of early ear development by integrating QTL mapping of inflorescence meristem (IM) activity with mature ear-related traits. In total, we identified 32 QTLs associated with ear development. Notably, our mapping results successfully recapitulated multiple previously reported domestication-related loci, including *KRN5.04* [63], and *TSH4* [13], thereby validating the robustness and genetic power of the TM population. More importantly, we uncovered a novel QTL cluster on chromosome 3, designated *qIM3*, which does not overlap with any known ear-related genes. This finding indicates that the regulatory network controlling maize ear morphology is considerably more complex than currently appreciated.

Previous studies on maize domestication have largely focused on genes acting at relatively late stages of inflorescence development, mainly affecting spikelet patterning, kernel row formation, and grain set after meristem differentiation [71]. In contrast, the identification of *qIM3* supports the existence of an additional regulatory layer functioning at earlier developmental stages, which modulates the initial size and activity of the inflorescence meristem (IM). By directly quantifying IM-related traits, including inflorescence meristem height (IMH) and width (IMW), we minimized confounding effects associated with developmental abnormalities. Especially, the female inflorescence meristem frequently exhibits partial apical masculinization at later developmental stages. Additionally, the late-stage environmental variation usually compromises the accuracy of mature ear traits such as ear length in teosinte introgression lines [72]. Consequently, IM-related traits serve as more direct and robust indicators of the genetic control of early ear development, thereby providing empirical support for a hierarchical and continuous genetic framework of maize ear domestication.

Consistent with this notion, QTLs controlling IM size showed extensive overlap with those affecting mature ear traits (Fig. 2), indicating a tight genetic coupling between early meristem activity and final ear morphology. In particular, the repeated co-localization of *qIM3* across multiple ear traits suggests that maintenance of IM activity represents a key genetic basis for ear development, supporting the notion that ear traits are partly determined at the IM stage. Notably, although these loci were largely shared, QTLs identified using IM-related traits exhibited narrower confidence intervals and more sharply defined peaks than those detected for mature ear traits, highlighting the higher resolution and robustness of IM-based genetic dissection. Moreover, *qKW* and *qEW* were co-localized with IM-associated QTLs at *qIM3* with a high degree of positional concordance, suggesting that this locus not only governs inflorescence meristem activity but also makes a substantial contribution to yield formation. Importantly, *qIM3* was identified as a pleiotropic locus that simultaneously regulates IM-related traits and mature ear characteristics, providing compelling evidence that early IM activity represents a key upstream determinant of ear architecture. Together, these results establish IM traits as powerful entry points for dissecting the genetic basis of complex ear-related phenotypes.

**Fig. 5:**
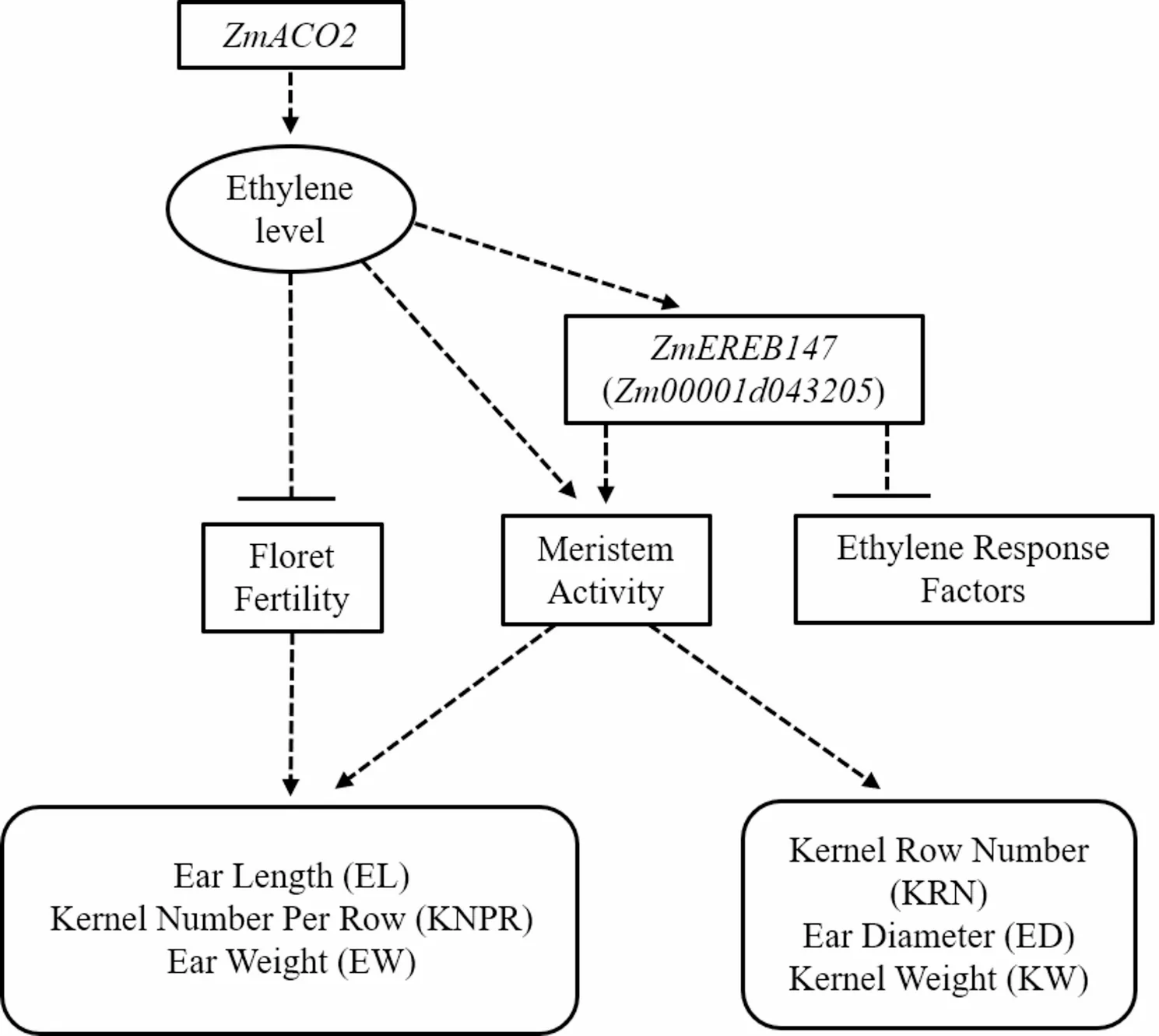
*ZmEREB147* (*Zm00001d043205*) regulates ear morphogenesis through the ethylene signaling response pathway in maize. This diagram illustrates the regulatory role of *ZmEREB147* within the ethylene signaling pathway and its impact on ear development traits in maize. *ZmACO2* modulates ethylene production and activates the downstream ethylene-responsive transcription factor *ZmEREB147*. Concurrently, *ZmACO2* acts to inhibit floret fertility, collectively shaping the yield. *ZmEREB147* participates in ethylene-mediated regulation of meristem development, thereby shaping maize ear architecture, including EL, KRN, ED, EW, and KW. Arrows indicate activation, and T-bars indicate inhibition

Ethylene is a key endogenous hormone regulating the transition from vegetative to reproductive growth in maize, and ethylene levels modulate flowering time and inflorescence meristem development, thereby optimizing plant architecture and root system development [73]. *ZmACO2*, functioning as an ACC oxidase, is the terminal key enzyme in the ethylene biosynthesis pathway. Simultaneously, *ZmACO2* positively regulates inflorescence meristem size while negatively affecting ear length, kernel number and yield in maize by regulating ethylene biosynthesis, indicating a dual regulatory role in balancing meristem maintenance and organ elongation [49]. *ZmEREB147* (*Zm00001d043205*)was identified as a differentially expressed gene associated with *ZmACO2* allelic variation. Specifically, in 2-mm ear primordia, its expression was significantly up-regulated in qEL7^*SL17*^ compared to qEL7^*Ye478*^ (log₂FC = 1.64, padj < 0.05), with expression levels of 99.2 and 31.7 FPKM, respectively. We analyzed the expression of *ZmEREB147* in developing ears (5‑mm stage) across 117 diverse inbred lines and correlated it with mature ear length. We observed a significant positive correlation (Pearson’s *r* = 0.45, *p* < 0.001; Fig. S3, Table S8). Collectively, these findings suggest that *ZmEREB147* is the most likely candidate gene for *qIM3*. The differential expression between maize and teosinte may modulate ethylene pathway-related gene expression, regulating the IM activity and ultimately leading to variation in mature ear traits. During the ethylene response pathway, *ZmACO2* enhances the expression of *ZmEREB147*, promoting meristem activity, thereby altering maturity-related traits. In addition, *ZmACO2* directly reduces floret fertility, which contributes to the yield traits variation. (Fig. 5)

Ethylene plays an important role in regulating plant developmental transitions and organ differentiation. *ZmACO2* encodes a key rate-limiting enzyme in ethylene biosynthesis and influences inflorescence architecture. By integrating transcriptome profiling, differential expression analysis of near-isogenic lines at *qIM3*, tissue-specific expression profiles in developing ears, and functional annotations, we identified *ZmEREB147* as the most promising candidate gene underlying *qIM3*. *ZmEREB147* encodes a nuclear-localized AP2/ERF transcription factor. *ZmACO2* can positively regulate the expression level of *ZmEREB147*. *ZmEREB147* is regarded as a downstream target, positively regulated by *ZmACO2*, and this regulation increases IM activity and ear-related traits. This implies that *ZmEREB147* links ethylene signaling with IM activity and ear architecture.

Based on these findings, we propose the *ZmACO2*-ethylene-*EREB147* regulatory module in which *ZmACO2* may act through *ZmEREB147* downstream of ethylene signaling to regulate IM development. (Fig. 5). Increased *ZmACO2* expression may enhance ethylene level, thereby activating *ZmEREB147*, negatively regulating the genes related to the ethylene response pathway. In addition, the higher ethylene levels may reduce the floret fertility, which is generally associated with increased final ear length and grain yield [49]. Ethylene may participate in cross-talk with other phytohormones in developing ears, and the coordinated regulation of genes involved in inflorescence development may also indirectly influence yield-related traits [74, 75]. *ZmEREB147* was identified as a differentially expressed gene associated with *ZmACO2*, showing lower expression in the long-ear allele qEL7^*Ye478*^ and higher expression in qEL7^*SL17*^. This indicates that *ZmEREB147* positively promotes meristem activity and acts downstream of *ZmACO2*-mediated ethylene signaling. Besides, *ZmEREB147* cooperates with the TOPLESS-like corepressor *REL2* to regulate the transcription of key flowering genes, thereby mediating inflorescence development and flowering time in maize [76]. *ZmEREB147* encodes an AP2/ERF transcription factor and acts as a key regulator in the ethylene‑accelerated leaf senescence signaling network under nitrogen deficiency [77]. Based on these observations, we propose that *ZmEREB147* modulates the strength of ethylene signaling, thereby promoting inflorescence meristem (IM) activity while buffering the inhibitory effects of ethylene on ear elongation. Collectively, these findings suggest that *ZmACO2* plays a dual and stage-dependent role during ear development: at early developmental stages, moderate *ZmACO2* expression enhances ethylene biosynthesis and activates *ZmEREB147*, facilitating the establishment of IM activity and ear length, with *ZmEREB147* serving as a key regulator balancing IM maintenance and ear axis elongation.

## Conclusion

In conclusion, our study underscores the critical importance of reliable QTLs for developing effective strategies to enhance grain yield in maize. We identified 32 consensus QTLs associated with ear development in a Teosinte-Maize introgression (TM) population. We predicted one candidate gene within the newly identified QTL interval. Notably, qIM3, a QTL controlling inflorescence meristem (IM) size, exhibited substantial overlap with QTLs affecting mature ear traits, revealing a tight genetic coupling between early meristem activity and final ear morphology. This work not only validates the practical utility of the newly identified QTLs for breeding but also provides a valuable strategy for improving complex traits in other crops.

## Supplementary Information

Supplementary Material 1: Figure S1. Developmental comparison between normal floret differentiation and apical masculinization of the ear. Figure S2. The ear phenotypes IMH and IMW of the NILs NIL-qIM3^Z58^ and NIL-qIM3^Teo^. Figure S3. Relationship between *ZmEREB147* expression (FPKM) and ear length.

Supplementary Material 2. Table S1. Linear Mixed Model, broad-sense heritability analysis, and descriptive statistics for Ear Length (EL), Ear Diameter (ED), Kernel Row Number (KRN), Inflorescence Meristem Height (IMH) and Inflorescence Meristem Width (IMW). Table S2. Descriptive statistics for Kernel Weight (KW) and Ear Weight (EW). Table S3. QTL mapping results of KRN in three environments. Table S4. QTL mapping results of EL in three environments. Table S5. QTL mapping results of ED in three environments. Table S6. QTL mapping results of IM in three environments. Table S7. Mean values of traits for each family line in each environment. Table S8. *ZmEREB147* expression (FPKM) and ear length (EL) in a diverse panel of 117 inbred lines.

## Data Availability

The raw RNA‑seq data generated in this study have been deposited in the NCBI Sequence Read Archive (SRA) under BioProject accession PRJNA1418938. The dataset is publicly available and can be accessed directly at https://www.ncbi.nlm.nih.gov/bioproject/PRJNA1418938.

## Abbreviations

ZmCCT10: Zm00001d024909
ZmCCT9: Zm00001d000176
FEA4: Zm00001d037317
TSH4: Zm00001d020941
UPA2: Zm00001d002562
ZmWUS1: Zm00001d001948
ZmWUS2: Zm00001d026537
TD1: Zm00001d014793
FEA2: Zm00001d051012
FEA3: Zm00001d040130
ZmCRN: Zm00001d042268
ZmACO2: Zm00001d020686
KRN2: Zm00001d002641
KRN4: Zm00001d052889
KRN5.04: Zm00001d016075
TGA1: Zm00001d049822
BA1: Zm00001d042989
BA2: Zm00001d003897
BIF2: Zm00001d031068
ZmPIN1a: Zm00001d044812
BAF1: Zm00001d045427
SPI1: Zm00001d044069
VT2: Zm00001d008700
