# Supplementary material for: Identification of a pleiotropic QTL and candidate genes underlying ear traits of maize (Zea mays L.) through controlling the activity of inflorescence meristem
Source: BMC Genomics. 2026 Jul 21;27:866. doi: 10.1186/s12864-026-13186-9 (PMC13678870; doi:10.1186/s12864-026-13186-9)
Supplement: Supplementary file 1 [file 12864_2026_13186_MOESM1_ESM.docx]

Figure S1. Developmental comparison between normal floret differentiation and apical masculinization of the ear.

(a) Normal floret differentiation in ears at 2 mm, 5 mm, and 1 cm stages.

(b) Ear at the 1-2 cm stage. The left shows a normally developing ear, whereas the right displays an ear with apical masculinization.

(c) Mature ears showing normal development (left) and apical masculinization (right).

Figure S2. The ear phenotypes IMH and IMW of the NILs NIL-qIM3^Z58^ and NIL-qIM3^Teo^

(a) Ear at the 5 mm stage. The left shows the ear tip of NIL-qIM3^Z58^, the right displays the ear tip of NIL-qIM3^Teo^.

(b) The mean IMH and IMW values of the NILs NIL-qIM3^Z58^ and NIL-qIM3^Teo^. Student's t test was applied to evaluate the statistical significance of differences. ***P < 0.001.

Figure. S3 Relationship between *ZmEREB147* expression (FPKM) and ear length. The top and right marginal plots show the distributions of FPKM and EL, respectively. The unit of ear length is centimeter (cm). The red line indicates the linear regression fit (FPKM ~ EL), showing the overall trend between ear length and gene expression. The orange shaded area represents the 95% confidence band of the regression line, illustrating the uncertainty of the fitted line. The Pearson correlation coefficient (r) and p‑value are displayed at the top left.
